# An evaluation of the ecological and environmental security on China’s terrestrial ecosystems

**DOI:** 10.1038/s41598-017-00899-x

**Published:** 2017-04-11

**Authors:** Hongqi Zhang, Erqi Xu

**Affiliations:** Key Laboratory of Land Surface Pattern and Simulation, Institute of Geographic Sciences and Natural Resources Research, Chinese Academy of Sciences, Beijing, 100101 P.R. China

## Abstract

With rapid economic growth, industrialization, and urbanization, various ecological and environmental problems occur, which threaten and undermine the sustainable development and domestic survival of China. On the national scale, our progress remains in a state of qualitative or semi-quantitative evaluation, lacking a quantitative evaluation and a spatial visualization of ecological and environmental security. This study collected 14 indictors of water, land, air, and biodiversity securities to compile a spatial evaluation of ecological and environmental security in terrestrial ecosystems of China. With area-weighted normalization and scaling transformations, the veto aggregation (focusing on the limit indicator) and balanced aggregation (measuring balanced performance among different indicators) methods were used to aggregate security evaluation indicators. Results showed that water, land, air, and biodiversity securities presented different spatial distributions. A relatively serious ecological and environmental security crisis was found in China, but presented an obviously spatial variation of security evaluation scores. Hotspot areas at the danger level, which are scattered throughout the entirety of the country, were identified. The spatial diversities and causes of ecological and environmental problems in different regions were analyzed. Spatial integration of regional development and proposals for improving the ecological and environmental security were put forward.

## Introduction

China is undergoing fast economic growth, industrialization, urbanization with huge environmental costs over a long period^1^. The excessive pursuit of gross domestic product (GDP) growth by over management and irrational development has heavily exploited natural resources and damaged environment conditions. This results in a series of serious ecological and environmental problems, such as water scarcity and contamination, air pollution, soil erosion, ecosystem degradation, and loss of biodiversity^2–4^. The ecosystem degradation and environmental pollution threaten and undermine the country’s economic and social growth, as well as domestic survival and development. Faced with the considerable ecological and environmental problems, is there an ecological and environmental security crisis, which indicated a hazardous situation causing the deleterious or potentially even disastrous consequences for humans^5–7^, in China’s terrestrial ecosystems? It calls for a systemic and comprehensive evaluation.

With different ecological and environmental problems in China, the evaluation of ecological and environmental security has progressively matured for the sustainable development and management of regional areas^8–10^. In particular, the evaluations were performed for water ecological security^11, 12^, land ecological security^8, 13, 14^, and urban ecological security^15, 16^. Thus far, limited studies have qualitatively or semi-quantitatively assessed the ecological and environmental security in China^2–4, 17^. The quantitative evaluation and its spatial distribution of ecological and environmental security are insufficient, which leads to ambiguous policy formulation and confounds implementation on a national scale.

Because the ecological and environmental security involves various definitions and is still a polysemous category^9, 18, 19^, we defined it as the characterization of the health status of ecosystems and the ability of ecosystem service supplies for humans. Until now, there has been no uniform and well-recognized indicator system. Faced with the complex and considerable ecological and environmental problems in China, there is a great difficulty in the evaluation of the ecological and environmental security status because of the unavailability of data and models. Our evaluation framework focused on the resource scarcity, ecosystem degradation, and environmental pollution, which were seen as the major ecological and environmental problems in China^2–4, 17^. Based on the different ecosystem elements, including four sub-ecosystems of hydrosphere, pedosphere, atmosphere and biosphere, this study identified the key indicators to build a comprehensive evaluation system, which was closely related to the ecological and environmental security status and above specific problems, and to assess the ecological and environmental security of China’s terrestrial system in around 2010.

## Results

### Water security evaluation

**Figure 1:**
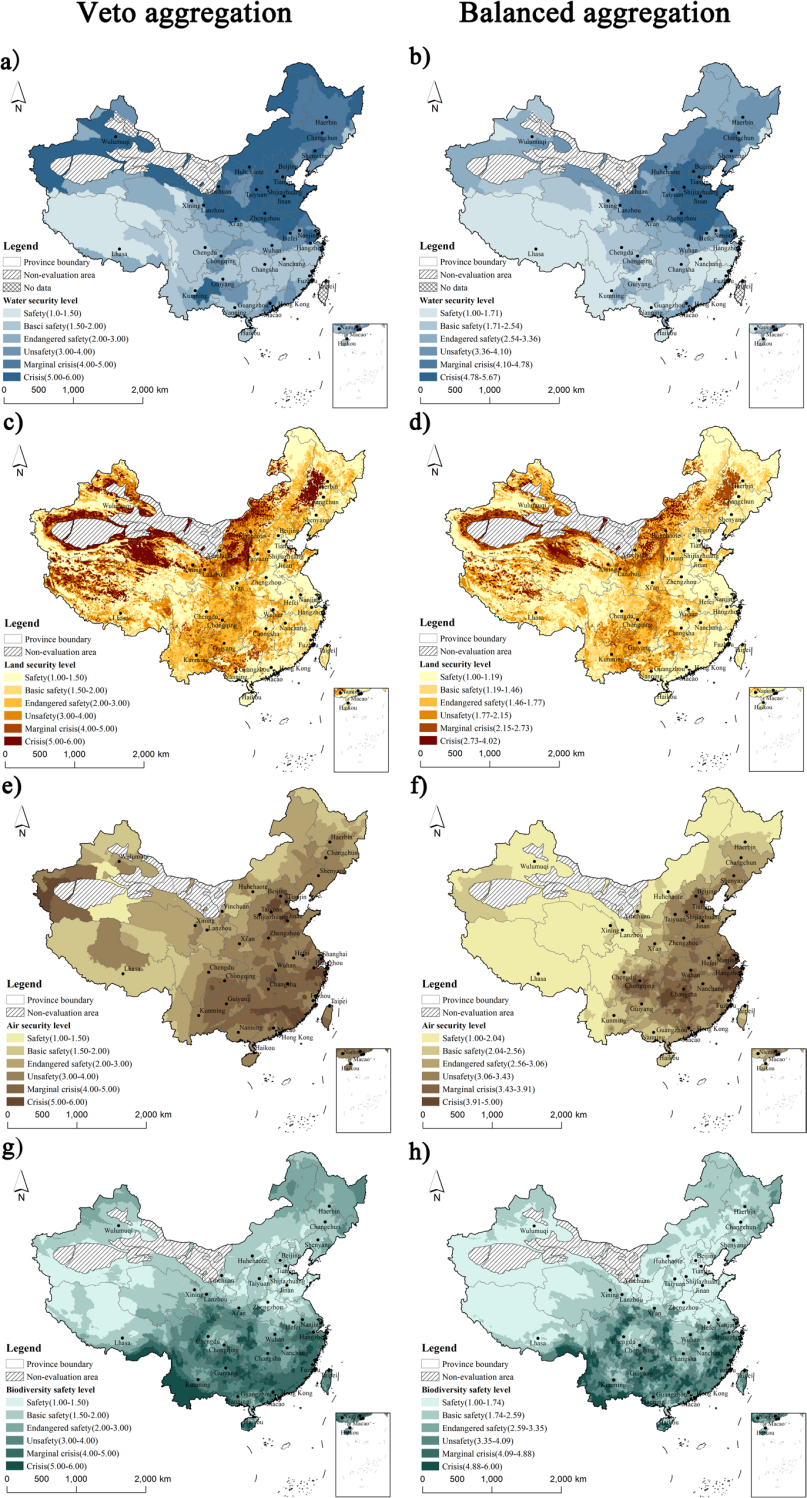
Spatial distribution of veto aggregation result including (**a**) water security in 2010; (**c**) land security in 2010; (**e**) air security in around 2010; (**g**) biodiversity security in around 2010; and balanced aggregation result including (**b**) water security in 2010; (**d**) land security in 2010; (**f**) air security in around 2010; (**h**) biodiversity security in around 2010. Maps were generated by ArcGIS version 10.1.0 (http://www.esri.com/software/arcgis). Note: Non-evaluation areas were at the extreme of degradation and cannot support human survival and development.

The veto aggregation focusing on the most serious security deficits and the balanced aggregation methods coordinating all indicators were used to comprehensively evaluate the security status for six classes, from the highest security class (safety) to the lowest security class (crisis). Results of water security in China are shown in Fig. 1a and b. Either veto aggregation or the balanced aggregation methods presented an obviously serious water security status in northern China; however, a relatively good water security status was obtained for southern China. Almost all areas of the entire northern China are classified into class_4_, class_5_, or class_6_. Veto aggregation methods indicated that area proportions of class_1_ to class_6_ are 14.75%, 20.37%, 16.58%, 7.56%, 8.92%, and 31.82%, respectively (Table 1). Over 40% of the areas were up to the danger level (marginal crisis or crisis classes), implying that there is at least one water security indicator up to the danger level, which indicates a serious water security status in China. Areas belonging to the crisis class of water security evaluation are chiefly located in the North China Plain with serious water scarcity and contamination; the adjacent mountains in Yunnan-Guizhou Plateau in southwestern China, the Hulun Buir Grassland, the Great Khingan Mountains, and Sanjiang Plain in northeastern China were identified as facing serious water contamination, and northwestern China region is faced with serious water scarcity. The results of the balanced aggregation method showed that area proportions of class_1_ to class_6_ are 34.19%, 15.56%, 22.16%, 13.00%, 8.12%, and 6.97%, respectively (Table 1). With the scattered distribution of evaluation scores and using Jenks natural break optimization, the North China Plain and its surrounding region were still classified in the crisis class in the balanced aggregation evaluation. Areas of high water security are chiefly located in the Tibet Plateau, Hengduan Mountainous Region in southwestern China, and hills and mountainous in Fujian, Jiangxi, Guangdong, and Guangxi Province.

**Table 1:** Area proportion of ecological and environmental security level (%).

Security level	Water security		Land security		Air security		Biodiversity security		Ecological and environmental security	
	Veto aggregation	Balanced aggregation	Veto aggregation	Balanced aggregation	Veto aggregation	Balanced aggregation	Veto aggregation	Balanced aggregation	Veto aggregation	Balanced aggregation
Safety	14.75	34.19	35.96	42.19	2.87	50.56	18.17	39.92	5.52	13.62
Basic safety	20.37	15.56	12.31	19.83	20.96	14.24	34.66	31.83	11.51	16.46
Early warning	16.58	22.16	21.39	15.8	26.53	11.38	19.73	9.45	16.83	20.65
Unsafety	7.56	13	13.06	10.36	27.78	14.09	12.79	10.43	21.97	20.14
Marginal crisis	8.92	8.12	5.58	8	18.1	6.64	9.79	4.3	16.83	20.77
Crisis	31.82	6.97	11.7	3.83	3.76	3.09	4.86	4.07	27.22	8.37

### Land security evaluation

Figure 1c and d show a similar spatial distribution between the results of veto aggregation and balanced aggregation methods. A relatively good land security was found in eastern China; however, a more serious land degradation and pollution threats were indicated in southwestern and northwestern China. Area proportions at the security level were the highest. Area proportions of safety to basic safety classes were 35.96% and 12.31% by the veto aggregation method, and 42.19% and 19.83% by the balanced aggregation method (Table 1). With the veto aggregation method, the area in the danger level of land security accounts for over 17% of the total evaluation area. It shows a diverse distribution of different land security evaluation indicators with significant geographical differentiation characteristics. Areas of serious land sandy desertification are mostly located on the edge of Gobi of the eastern Xinjiang Province, Taklimakan Desert, the Badain Jaran Desert, Tenggeli Desert in northwestern China, and the north of Tibet Plateau. Areas with the serious soil saline-alkali are mostly located on the edge of the Tarim Basin, the Qaidam Basin in northwestern China, the Hunshandake Sandy Area in northern China, the Songnen Plain in the northeastern China, and the north of Tibet Plateau. Although there are great improvements in the soil conservation in the Loess Plateau in western China, it still presents the most serious soil water erosion threat nationwide. The rocky karst desertification occurs in the southern karst areas, and areas of the most serious desertification are located in the adjacent mountains in Guizhou, Yunnan, and Guangxi Province. Areas of the serious heavy metal pollution are mostly in densely-populated cities and developed mining areas of the Hunan, Guizhou, Yunnan, and Guangxi Provinces. Areas with relatively good land security are mostly located in the eastern coastal areas, the Great Khingan and Changbai Mountains in northeastern China.

### Air security evaluation

Air security evaluations are shown in Fig. 1e and f. The figures show a more serious air security situation in the developed areas within mid-eastern China based on the two aggregation methods. Veto aggregation method indicates an unoptimistic air security status in China, where areas at safety and basic safety classes only accounted for 2.87% and 20.96% in the total evaluation areas, respectively (Table 1). This is may be attributed to the relatively high PM_2.5_ pollution in China, and even the PM_2.5_ pollution in part of the regions of Xinjiang and Tibet Province were up to a level indicative of insecurity. In detail, areas at the danger level for four air security evaluations indicator are mostly located in mid-eastern China but with a certain heterogeneously spatial distribution, as follows: areas at the danger level of inorganic nitrogen wet deposition exist, as does the pH level in acid rain located in wide areas in the southern China region and Sichuan Basin in southwestern China. The most serious PM_2.5_ pollution sites were mostly located in the North China Plain and the Middle and Lower Yangtze Plain in eastern China. Further, areas of the high carbon emission are located in the North China Plain and Lower Yangtze Plain in eastern China. The balanced aggregation method presented an obviously spatial distribution of a gradient increase from the west to east. Areas at the danger level of air security evaluation are mostly located in the Beijing-Tianjin-Hebei region in northern China, Middle and Lower Yangtze Plain in eastern China, and Sichuan Basin in southwestern China.

### Biodiversity security evaluation

Similar spatial distributions of threatened plants and animals result in a similar spatial distribution between the veto aggregation and balanced aggregation methods (Fig. 1g and h). Thus, the figures show a more serious biodiversity security problem in the south of China than that in the north of China. Almost all the wide areas in southern China are classified to the class_4_, class_5_, or class_6_. Except for the Great Khingan and Changbai Mountains in northeastern China, and the Altai Mountains in northwestern China; other regions in northern China are classified to class_1_ or class_2_. Area proportions at the security level are the highest, with over 50% of areas classified by the safety or basic safety classes, with 52.83% by the veto aggregation and 71.75% by the balanced aggregation method (Table 1). Area proportions at the marginal crisis and crisis classes are 9.79% and 4.86% by the veto aggregation, compared to the 4.30% and 4.07% areas by the balanced aggregation method (Table 1). Areas at the danger level present a relatively scattered distribution, including the Hengduan Mountains in southwestern China, hills in southern Yunnan Province, adjacent Mountains of Chongqing Municipality and Hubei Province, adjacent Mountains of Zhejiang Province and Fujian Province, the Nanling Mountains in southeastern China.

### Ecological and environmental security evaluation

**Figure 2:**
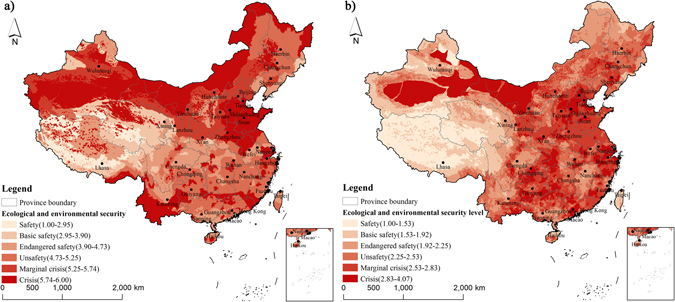
Spatial destitution of ecological and environmental security in China (**a**) veto aggregation method in around 2010; (**b**) balanced aggregation method in around 2010. Maps were generated by ArcGIS version 10.1.0 (http://www.esri.com/software/arcgis). Note: Non-evaluation areas, which were at the extreme of degradation and cannot support human survival and development, did not need to be evaluated and were labeled as the “crisis” class.

Figure 2 shows the final ecological and environmental security evaluation in terrestrial ecosystems of China. With different strategies and emphases of two methods, different numerical values and spatial distribution of evaluation results are shown. The veto aggregation score yielded a mean value of 4.83 and a standard deviation of 1.13, ranging from 1.00 to 6.00. In contrast, the balanced aggregation score yielded a mean value of 2.19 and a standard deviation of 0.51, ranging from 1.00 to 4.03. Areas at the danger level are the highest, which accounted for 43.05% of the total evaluation area by the veto aggregation method. In contrast, areas at the insecurity level are the highest by indication of the balanced aggregation method, which accounted for 40.79% of the total evaluation area (Table 1). Moreover, the evaluation scores by the veto aggregation method which are larger than 4.00 and 5.00 accounted for 70.09% and 44.00% of the evaluation area, which means that at least one security evaluation indicator is up to the crisis and marginal crisis classes in 70.09% and 44.00% of the evaluation area, respectively. Only 16.03% and 30.08% by the veto aggregation and balanced aggregation methods of the evaluation areas were classified to the security level. Additionally, the insecure ecological and environmental areas, including the non-evaluation (extreme of degradation and cannot support human survival and development), insecurity level and danger level, account for 84.52% and 72.85% of the national territorial area by the veto aggregation method and the balanced aggregation method, respectively. Both of the two aggregation methods showed a relatively serious ecological and environmental security crisis in China.

Integrating diverse spatial distribution of four aspects for the final ecological and environmental security evaluation, both the aggregation methods presented an obviously spatial variation of the security evaluation results (Fig. 2). The result showed that areas at the security level are mostly located in the Tibet Plateau, Tianshan Mountains and Altai Mountains in northwestern China, the Great Khingan Mountains in northeastern China, Hainan Island, and Taiwan Island. Areas at the insecurity and danger level are widely scattered in China. Figure 2 shows that areas of serious ecological and environmental security are located in either the developed eastern regions or the underdeveloped central and western regions in China. Overlaying Fig. 2a and b, the hotspot areas at the danger level, which were obtained by focusing on the limited security indicators by the veto method and integrating the compound security problems by the balanced aggregation method, were identified as follows: Gobi in Eastern Xinjiang, Taklimakan Desert, the Badain Jaran Desert, and Tenggeli Desert in northwestern China; Hunshandake Sandy Area, and North China Plain in Northern China; Lower Yangtze Plain in eastern China; adjacent hills and mountains in Yunnan-Guizhou Plateau, adjacent Mountains of Chongqing Municipality and Hubei Province in southwestern China; Pearl River Delta in southeastern China.

## Discussion

### Joint spatial distribution of ecological and environmental security

Using the veto aggregation and balanced aggregation methods, this study conducted a spatial evaluation on the ecological and environmental security in terrestrial ecosystems of China based on 14 security indicators. The GIS maps visualized the spatial distribution and identified the hotspot areas of water, land, air, biodiversity, to determine the final ecological and environmental security. According to the joint spatial distribution of all security evaluation indicators, we found that four aspects of ecological and environmental security have obviously different spatial distributions but a relatively similar distribution for several security indicators (Fig. 1). The problems of environmental pollution, including water pollution, soil heavy metal pollution, and air pollution, are primarily located in the mid-eastern China, especially the coastal eastern regions. The problems of land degradation, including soil water erosion, land sandy desertification and karst rocky desertification, are primarily located in the western China. Moreover, the problem of water scarcity is primarily located in the northern China. In contrast, the problem of threat to biodiversity is primarily located in the southern China. All of these may be attributed to the regional distribution of the resource abundance, physical geography characteristics, and human disturbance in China^20^. Physical geographical conditions determine the geographical distribution of climate, water, land, and biological resources in China. For example, the spatial distribution of land desertification status in the northwest is controlled by climate change and geomorphological processes even though human impacts have undeniably exacerbated these effects^21^. The land rocky desertification almost occurs in southwestern regions with the karst landscape^22^. In contrast, environmental pollution mainly originates from the strong anthropogenic disturbance, which resulted in a more serious pollution status in the developed eastern region^23, 24^.

### Uncertainty and of security evaluation results

With the difficulty and time-lag in data collection, it should be noted that the timeframes of datasets would potentially limit our results with the possibility of changes in recent years, since data were collected in around 2010 (Table 2). China has implemented large-scale environmental management and ecological restoration projects^4, 25, 26^, and the security status of China improved locally compared to our results. However, the ecological and environmental security did not change dramatically at the national scale and was still not optimistic^27^. For example, the PM_2.5_ pollution was still serious in 2016 with even a 7% increase of PM_2.5_ concentration in November of 2016^1^. Anyway, our results presented a spatially-explicit distribution to show the serious security issues (Fig. 2) and provided the effective information about the ecological and environmental security of China’s terrestrial ecosystems.

**Table 2:** Evaluation indicators of the ecological and environmental security.

	Evaluation indicator	Indicator definition	Data processing	Data resolution	Data sources	Data time
Water security	Water resources per capita (m^3^ per capita)	W/P W: Total water resource; P: Total population	Collection, spatial assignment and calculation	Second-level China’s Water Resources Zones	National integrated water resources planning^1)^	2010
	Utilization ratio of water resources (%)	(WS-WO + WI)/W × 100% WS: Water supply; WO: Water transferred out; WI: Water transferred in; W: Total water resource	Collection, spatial assignment and calculation	Second-level China’s Water Resources Zones	National integrated water resources planning^1)^	2010
	Proportion of I to III kinds of water (%)	L_(I to III)_/L_(All)_ × 100% L_(I to III)_: River lengths of I, II, III water quality; L_(All)_: Total evaluation river lengths	Collection, spatial assignment and calculation	Second-level China’s Water Resources Zones	National integrated water resources planning^1)^	2010
Land security	Water soil erosion (t/(hm^2^ · a))	R × K × LS × C R: rainfall erosivity factor; K: soil erodibility factor; LS: topographic factor; C: vegetation cover factor USLE model^51^	R: Daily Rainfall Erosivity Model^52^; K: Erosion/Productivity Impact Calculator^53^; LS: piecewise function based on the slope length and gradient^54–56^; C: assignment based on vegetation cover referring to literatures^57^	1 km * 1 km	Daily Rainfall data from 583 meteorological stations from (http://data.cma.cn/); Soil properties from (http://westdc.westgis.ac.cn/); SRTM DEM from (http://www.gscloud.cn/); MODIS NDVI (MOD 13A3) from https://ladsweb.nascom.nasa.gov/data/; Land use map from http://www.globallandcover.com/	2010
	Land sandy desertification (%)	Bare sand land proportion	Extraction from the land use map in 2010	1 km * 1 km	Resources and Environment, Chinese Academy of Science Data Center (http://www.resdc.cn/)	2010
	Soil saline-alkali (%)	Saline-alkali land proportion	Extraction from the land use map in 2010	1 km * 1 km	Resources and Environment, Chinese Academy of Science Data Center (http://www.resdc.cn/)	2010
	Karst rocky desertification	Visual interpretation using remote sensing images based on the bedrock exposure, vegetation and soil cover	Artificial digitization and projection	100 m * 100 m	Raw data from the China karst rocky desertification state bulletin^2)^	2011
	Soil heavy metal pollution	Poorest Classification of As, Cr, Cd, Hg, and Pb using the standards for soil environmental quality^3)^	Artificial digitization. Details were described in the paper^58^	2 km * 2 km	Raw data from the paper^58^	2011
Air security	Inorganic nitrogen wet deposition (kg · ha^−1^ · a^−1^)	Inorganic nitrogen wet deposition per hectare per year	Kriging interpolation of 144 monitoring sites data. Details of data processing were described in the paper^59^	10 km * 10 km	Data sources from published sources and National Acid Deposition Monitoring Network were described in the paper^59^	2000–2010
	Carbon emission (gC · m^−2^)	$\frac{{\sum }_{l}^{i}(e{c}_{i}\times {e}_{i}/T{e}_{i})+cc\times c/Tc}{A}$ *ec*_*i*_: carbon emissions of energy consumption; *e*_*i*_: standardized energy consumption of province in China, including coal, oil, and natural gas; *Te*_*i*_: total standardized energy consumption of China; *cc*: carbon emissions of cement production in China; *c*: cement production of province; *Tc*: total cement production of China; A: Province area	Collection, spatial assignment and calculation. The standardized energy consumption calculated by the consumption of coal, coke, crude oil, fuel oil, gasoline, kerosene, diesel and natural gas and the conversion Coefficient of standard coal unit. Details were described in the paper^60^	Province unit	Carbon emissions (*ec*_*i*_ and *cc*) from the Carbon Dioxide Information Analysis Center; Energy consumption data from China Energy Statistical Yearbook^4)^; Cement production data from China Statistics Yearbook^5)^	2000–2010
	Annual PM_2.5_ (μg · m^−3^)	${\sum }_{1}^{365}$ C_i_(PM_2.5_)/365 C_i_(PM_2.5_): Daily PM_2.5_ mass concentration	Collection, spatial assignment and calculation	Municipal unit	China National Environmental Monitoring Center	2014
	pH in acid rain	${\sum }_{1}^{365}$ pH_i_/365 pH_i_: Daily pH of rain	Collection, spatial assignment and Kriging interpolation of 252 acid rain monitoring sites	10 km * 10 km	China National Environmental Monitoring Center	2010
Biodiversity security	Threatened plants (type/county)	NP + NS + NC + NM NP: number of threatened Pteridophyte; NS: number of threatened Spermatophyte; NC: number of threatened Cyonophyta; NM: number of threatened Mycophyta	Artificial digitization. Details were described in the paper^39^	County unit	Raw data from the paper^31^	2007
	Threatened animals (type/100 km^2^)	NM + NB + NA NM: number of threatened Mammal per unit; NB: number of threatened bird per unit; NA: number of threatened amphibian per unit	Data masking and raster calculation.	10 km * 10 km	Mapping the World’s Biodiversity^6)^	2010

Note:

^1)^Ministry of Water Resources of People’s Republic of China, National integrated water resources planning, 2010. (in Chinese).

^2)^State Forestry Administration of People’s Republic of China, 2012, China karst rocky desertification state bulletin. (in Chinese).

^3)^China National Environmental Protection Agent, 1995. GB15618-1995: Environmental Quality Standard for Soil. China Environmental Science Press, Beijing (in Chinese).

^4)^National Bureau of Energy Statistics Division, General Affairs Department of the National Energy Bureau of China Energy Statistical Yearbook. Beijing: China Statistics Press, 2000–2010. (in Chinese).

^5)^China Statistics Yearbook of People’s Republic of China, National Bureau of Statistics, Beijing: China Statistics Press, 2000–2010. (in Chinese).

^6)^Available online: http://www.biodiversitymapping.org.

The results presented a certain degree of difference from the spatial distributions of the security evaluation results obtained using the two aggregation methods (Figs 1 and 2), which can help to explain different aspects of results^28, 29^. The veto method focused on the most serious security evaluation indicator and can also identify the limited security evaluation indicator at each grid, which presented a more obviously spatial diversity. In contrast, the balanced aggregation method can comprehensively integrate all the indicators and give a relatively even evaluation. This method permitted compensation between indicators, that is, a deficit in one indicator could be offset by a surplus in another^28^, which presented a more scattered spatial distribution (Figs 1 and 2). It is worth noting that limitations of the two methods lead to some uncertainties. For example, the water pollution in the Great Khingan Mountains and Sanjiang Plain in northeastern China are serious, but other indicators are classified at the security level, which result in a higher class in the veto aggregation method but a lower class in the balanced aggregation method (Fig. 2). Similar differences can be found in the edges of deserts in Northwestern China region with serious land insecurity but relative security in other indicators; moreover, the hills in southern Yunnan Province show a danger level of biodiversity security but relatively secure classes in other indicators. In contrast, the Pearl River Delta was classified into relatively secure classes in the sole evaluation indicator by the veto aggregation method as opposed to the high security class obtained by the balanced aggregation method. This result indicated that various aspects of ecological and environmental security in the Pearl River Delta were not up to the crisis class; however, they remain at a relatively serious security class. Therefore, we call for a combined evaluation of the two methods to explore the ecological and environmental security crisis in China, to decrease the uncertainty originating from singular evaluation.

Scale was an important and inevitable issue in the evaluation processes^28^. A uniform grid of 10 km × 10 km, which was basically determined by the mean unit size of all indicators at different scales, was used to execute the data assimilation in our study. To examine the scale effects of the uniform grid on evaluation results, we used the grid of 5 km × 5 km and 20 km × 20 km to re-execute the evaluation process. It found the almost similar mean values and standard deviation values for two aggregation methods. For veto aggregation scores, the mean values of 5 km × 5 km, 10 km × 10 km, and 20 km × 20 km were 4.84, 4.83, and 4.83, respectively; and the standard deviations at three grid sizes were 1.15, 1.13, and 1.12, respectively. Similar results were found in the balanced method. Comparing results at the three grid sizes, the grid of 5 km × 5 km generated the most scattered spatially distribution of security evaluation result. Although a coarse grid size would result in a smooth result, using scaling transformations, the evaluation scores were transformed to the same frequency histogram from raw evaluation data. Therefore, no evidence for scale effects in determining the degree of ecological and environmental security results was found. We thought that the grid size of 10 km × 10 km provided a reasonable evaluation result at the national scale.

### Causes of the ecological and environmental security deterioration

Either individual or comprehensive evaluation results indicated a relatively serious ecological and environmental security in China. These can be attributed to natural factors but also to anthropogenic factors, including the historical legacy, but also the irrational use and destruction over the past century. China, consisting of three topography steps with a high proportion of mountains, are majorly located in the eastern Asian monsoon climate zone^20^. This resulted in the wide distribution of ecologically fragile regions, including the arid desert, the alpine region, the Loess Plateau region, karst regions and agro-pastoral zone^21, 30, 31^. The basically natural factors and physical geographical characteristics caused the resource deficiencies and ecosystems fragility. With the population pressure, economic development, and rapid urbanization, the predatory management and excessive pursuit of the GDP target accelerated resource waste, land desertification, and environmental pollution^32^. Moreover, the science and technology of ecological restoration and environmental protection is lagging, which increases the difficulty in helping to solve important ecological problems and to provide effective knowledge and technology reserves for the state^27^.

### Integrated regional development to improve ecological and environmental security

Faced with the current ecological and environmental security status in China, a solution calls for the spatially regional integration and development strategies to relieve the pressure on the environment and to implement ecological restoration and environmental governance for different ecological and environmental security problems. We suggest that eastern coastal areas should be committed to make efforts to govern the environmental pollution, including air, water, and soil pollution. In addition, a solution calls for a transformation of economic development and an acceleration of industrial restructuring and upgrading. Central regions have the advantage of linking the eastern coastal areas and western areas, as well as geographical advantages and the flat topography to develop modern agriculture. Further, we suggest that the central regions focus on the development of agricultural modernization, and also, on the ecological restoration and environmental governance concentrated on the comprehensive management of major rivers, remediation of soil metal heavy pollution, and soil conservation. For the western region, which has ecologically fragile areas, the protection of the ecological environment should be made prominent. Under this premise, we suggest an appropriate integration of ecological migrants, the economic development in key areas, and the development of natural resources, step-by-step. With the spatial development and integration of the resource-ecology-economy, we look forward to improve the ecological and environmental security and to promote the sustainable development of society in China.

In conclusion, our study assessed the spatial distribution, visualized different security aspects, and identified hotspots of ecological and environmental security in terrestrial ecosystems of China On the national scale. Based on 14 security evaluation indicators for the water, land, air, and biodiversity security, with area-weighted normalization and scaling transformation approaches, this study used the veto aggregation and balanced aggregation method to comprehensively assess the ecological and environmental security status of China’s terrestrial ecosystems. Both these methods indicated a relatively serious ecological and environmental security situation, but presented an obviously spatial variation of the six security evaluation classes. Areas of unsafe ecological and environmental security, including the non-evaluation, insecurity level and danger level, account for 84.52% by the veto aggregation method and 72.85% by the balanced aggregation method of the China’s territorial area. Areas of serious water security issues are mostly located in northern China, serious land security issues in mid-western China, serious air security issues in eastern China, serious biodiversity security issues in southern China. With the final security evaluation, areas at the healthy security level are mostly located in the Tibet Plateau, Tianshan Mountains, and Altai Mountains in western China, the Great Khingan Mountains in northeastern China, Hainan Island, and Taiwan Island. Areas at the danger level are widely scattered in China, and corresponding hotspots were identified. The integration of regional development was suggested to improve the ecological and environmental security deterioration in China.

## Method

### Evaluation index system of ecological and environmental security

A framework for selecting indicators should take the goals defining, context understanding, and stakeholders identifying into consideration to develop criteria for indicator selection^33^. In consultation with other researchers and policy makers, this study built the ecological and environmental security framework of indicator selection, which comprehensively characterized the health status of ecosystems, the ability of ecosystem service supplies for humans, and the major current ecological and environmental problems in China^2–4, 17^. Considering the system complexity and data availability at the national scale, our framework selected limited indicators from four basic sub-ecosystems (hydrosphere, pedosphere, atmosphere and biosphere).

Finally, we collected and classified the 14 indicators, such as water security, land security, air security, and biodiversity security in Table 2, for the purpose of building the ecological and environmental security evaluation indicator system. Water security includes water scarcity and contamination^34^. Land security consists of different land degradation types (soil water erosion, land sandy desertification and karst rocky desertification in different regions) and soil heavy metal pollution^35, 36^. Air security is heavily characterized by the nitrogen deposition, carbon emission, acid rain, and PM_2.5_ pollution^37, 38^. Biodiversity security is characterized by the status of threatened plant and threatened animal life^39, 40^. Details of how all the security evaluation indicators were collected or calculated are shown in the Table 2.

Different data were primarily evaluated into different categories based on the corresponding grading standard from the professional sectors and previous studies (Table 3). All of the grading standards were used to indicate different security states of the evaluation subsystem but with different descriptive ways, i.e. the poor, dangerous security for the low level; the unsafe, worsening, threating security for the medium level; the general, ideal, safe security for the high level^41–43^. To build a unified security evaluation system, the different numbers of indicators and grading obtained from different studies were grouped in three levels (A: security; B: insecurity; and C: danger) and six sequential classes (class_1_: safety; class_2_: basic safety; class_3_: early warning; class_4_: unsafety; class_5_: marginal crisis; class_6_: crisis). Reference to previous study^41–43^, meanings of six security classes were shown in Table 4, which indicated the relative safeguard degree and expectation state of evaluation subsystem, and the reliability of prevent imperfect and threating event to happen based on professional sectors and document retrievals.

**Table 3:** Grading standards of evaluation indicators for the ecological and environmental security.

	Evaluation indicator	Grading basis	Grading standard					
			Security (A)		Insecurity (B)		Danger (C)	
			Safety (1)	Basic safety (2)	Early warning (3)	Unsafety (4)	Marginal crisis (5)	Crisis (6)
Water security	Water resources per capita (m^3^ per capita)	Gong, *et al*.^61^	>3 000	2 000–3 000	1 000–2 000	500–1 000	<500	
	Utilization ratio of water resources (%)	Gong, *et al*.^61^	<10	10–20	20~40	40~70	>70	
	Proportion of I to III kinds of water (%)	ref. 61	>90	70–90	60–70	50–60	40–50	0–40
Land security	Water soil erosion (t/(hm^2^ · a))	Standards for Grading of Soil Erosion (SL190-96)^1)^	<5	5–25	25–50	50–80	80–150	>150
	Bare sand land proportion (%)	Xue, *et al*.^62^, Li, *et al*.^63^	No sandy desertification	0–5	5–25	25–50	50–70	70–90
	Saline-alkali land proportion (%)	Yang, *et al*.^64^	No salinization - alkalization	0–10	10–20	30–50	50–70	70–90
	Karst rocky desertification class	Chinese karst rocky desertification state bulletin^2)^	No KRD	Potential KRD	Slight KRD	Moderate KRD	Severe karst KRD	Extremely severe KRD
	Soil heavy metal pollution class	Yang, *et al*.^58^ based on the Soil environmental quality standard^3)^	Clean or relatively clean		Normal	Polluted	Moderately to heavily polluted	
Air security	Inorganic nitrogen wet deposition (kg · (hm^2^ · a)^−1^)	Jia, *et al*.^59^	<5	5–10	10–20	20–25	25–30	>30
	Carbon emission (gC · m^−2^)	Yue, *et al*.^60^	<180		190–500	550–1020	1450–2000	>2 000
	Annual PM_2.5_ (μg · m^−3^)	National Ambient Air Quality Standard (GB 3095–2012)^4)^, Wang, *et al*.^24^	0–10	10–35	35–50	50–75	75–100	>100
	pH in acid rain	National Ambient Air Quality Standard(GB 3095–2012)^65^	≥5.60		5.00–5.60	4.50–5.00	<4.50	
Biodiversity security	Threatened plants (type/county)	Zhang and Ma^39^	0	1–4	5–8	9–12	13–19	20–40
	Threatened animals (type/100 km^2^)	Zhang, *et al*.^40^	0–4	5–8	9–12	13–16	17–19	20–37

Note:

^1)^Ministry of Water Resources of the People’s Republic of China, 1997, SL190-96 Standards for Grading of Soil Erosion. (in Chinese).

^2)^State Forestry Administration of People’s Republic of China, 2012, China karst rocky desertification state bulletin. (in Chinese).

^3)^China National Environmental Protection Agent, 1995. GB15618-1995: Environmental Quality Standard for Soil. China Environmental Science Press, Beijing. (in Chinese).

^4)^Ministry of Environmental Protection, 2012, National Ambient Air Quality Standard (GB 3095-2012). (in Chinese).

**Table 4:** Explanations on ecological and environmental security classes.

Security class	Class name	Index Characteristic
1	Safety	Subsystem stays a healthy state, functions well and completely, has strong resilience, ecological and environmental problems are not obvious, there are little ecological and environmental disasters.
2	Basic safety	Subsystem function properly, presents a relatively strong resilience, there is several ecological and environmental problems, disasters are not significant.
3	Early warning	Subsystem functions with potential risks, there are several ecological and environmental problems, ecological disasters occur sometimes, it vulnerability calls for restoration of the subsystem.
4	Unsafety	Subsystem is damaged at a certain extent, functions unsustainably and insufficiently, has an obviously degraded resilience, the restoration of the subsystem has certain difficulties.
5	Marginal crisis	Subsystem functions poorly, ecological restoration and reconstruction are difficult, ecological and environmental problems and disasters occur a lot.
6	Crisis	Subsystem is damaged seriously, causes the deleterious or potentially even disastrous consequences for humans.

Note: Explainations of six security classes were referenced to previous study^41–43^, which indicated different security states of the evaluation subsystem but with different descriptive ways.

Here, the lowest security class is named as the “crisis”. As modern concept of “crisis” has a distinguished history, ably recalled by Jim O’Connor^44^ and discussed by previous studies^6, 45^. This study defined it as a hazardous situation causing the deleterious or potentially even disastrous consequences for humans^5–7^. Not all processes or subsystems have well-defined thresholds of “crisis”^7^, then we identified the “crisis” class as the extreme lack of resource scarcity, the most serious ecosystem degradation, or the most hazardous environment pollution. The grading thresholds of “crisis” class were quantitatively determined by the corresponding grading standard (Table 3). For example, the value of 150 (t/(hm^2^ · a)), which indicated the most serious water soil erosion based on Standards for Grading of Soil Erosion (SL190-96)^2^, then was grouped as the crisis threshold of the water soil erosion.

It should be noted that all the indicators can be grouped as in the three levels, but only several indicators can be grouped in six classes. This is determined by the professional grading standard of the security evaluation indicator. Moreover, the evaluations in this study did not include the Gobi in Eastern Xinjiang, Taklimakan Desert, the Badain Jaran Desert, and Tenggeli Desert in northwestern China, areas which are at the extreme of degradation and cannot support human survival and development. These areas were labeled as non-evaluation areas, accounting for 9.72% of the national territorial area (Fig. 1). Thus, the area proportional to the evaluation area is 90.28% of China’s territorial area.

### Comprehensive evaluation of ecological and environmental security

Diverse evaluation data were collected and quantified in different units and at different scales, for example, water scarcity was assessed at the Second-level of China’s Water Resources Zones, the soil erosion from water was characterized by the raster units of 1 km × 1 km, the karst rocky desertification data were collected at the raster units of 100 m × 100 m, and the PM_2.5_ concentration data were collected at the municipal unit. To comprehensively assess the ecological and environmental security of the terrestrial ecosystems in China, we used a uniform grid of 10 km × 10 km to calculate the corresponding normalization scores for different security evaluation indicators. Finally, fourteen indicators from four aspects were aggregated by two aggregation methods to evaluate the ecological and environmental security at each uniform grid.

#### Area-weighted normalization score

We assigned evaluation scores of six security classes as 1, 2, 3, 4, 5, and 6 from class_1_ to class_6_ for each security evaluation indicator at the unit. If one certain level is not included in the class, the scores of two classes were assigned the mean value of two corresponding classes. For example, the level A was not included in the classes of safety and basic safety for the evaluation of nitrogen deposition, therefore, both A_1_ and A_2_ were assigned as 1.5. Then, we used the uniform grid to calculate the area-weighted evaluation normalization score^46, 47^ by the following equation:

1 $${S}_{i}^{j}=\sum _{k=1}^{{n}_{i}^{j}}{S}_{ik}^{j}\times {A}_{ik}^{j}$$

where ${S}_{i}^{j}$ denotes the area-weighted evaluation sore at each uniform grid, *i* is the serial number of security evaluation indicators, and *j* is the serial number of the uniform grid; ${S}_{ik}^{j}$ denotes the score of class *k* of security evaluation for *i* indicator at *j* grid; *k* is the class of security evaluation indicators ranging from 1 to ${n}_{i}^{j}$; ${n}_{i}^{j}$ indicates the class number of *i* indicator; ${A}_{ik}^{j}$ denotes the area of the *k* class of *i* security evaluation indicator in the *j* grid. The range of ${S}_{i}^{j}$ is from 1.00 to 6.00. A higher ${S}_{i}^{j}$ implies a more serious status for the corresponding security evaluation indicator. If partial areas were not evaluated using a certain indicator because of data unavailability, the given areas were excluded in the calculation of the equation (1).

#### Scaling transformation method

With the area-weighted evaluation normalization, security evaluation indicator data at different resolutions were unified on the same grid. This, however, results in a statistical bias of scaling up or down. In particular, the evaluation scores of security evaluation indicators with fine resolutions would be smoothed when scaling up to a coarser resolution. Therefore, based on the idea of histogram transformation in image enhancement^48, 49^, we used the frequency histogram equalization to re-calculate the normalization scores from the raw data. Taking the indicator including six classes as an example, the scaling transformation of normalization scores were realized by the following steps. First, the area proportions of six security evaluation classes using the raw data were calculated to generate a frequency histogram with six classes (scores of class_1_ to class_6_ for 1, 2, 3, 4, 5, and 6). Second, we calculated piecewise normalization scores ${S}_{i}^{j}$ of six interval ranges, of which area proportions were consistent with area proportions of six classes in the histogram from step 1, i.e. (X_1_, X_2_], (X_2_, X_3_], (X_3_, X_4_], (X_4_, X_5_], (X_5_, X_6_]. Third, six interval ranges of normalization scores were transformed to the scaling interval ranges of 1, (1, 2], (2, 3], (3, 4], (4, 5], (5, 6], which are consistent with six interval ranges calculated from step 2. ${S}_{i}^{j}$ at the interval range of (X_1_, X_2_] were assigned 1. Other normalization scores were calculated at every pair of interval ranges by the following equation:

2 $$\frac{{S}_{ik}^{j^{\prime} }-{X}_{{\rm{\min }}}^{^{\prime} }}{{X}_{{\rm{\max }}}^{^{\prime} }-{X}_{{\rm{\min }}}^{^{\prime} }}=\frac{{S}_{ik}^{j}-{X}_{{\rm{\min }}}}{{X}_{{\rm{\max }}}-{X}_{{\rm{\min }}}}$$

where ${S}_{ik}^{j^{\prime} }$ is the scaling transformation score, *i*, *j*, and *k* are the same symbol in the equation (1); *X*_max_ and *X*_min_ mean the upper and lower limits of interval ranges from normalization scores in step 2; ${X}_{{\rm{\max }}}^{^{\prime} }$ and ${X}_{{\rm{\min }}}^{^{\prime} }$ denote the corresponding upper and lower limits of the scaling interval ranges, respectively. Taking the ${S}_{i}^{j}$ at the interval range of (X_3_, X_4_] as an example, it can be calculated as follows:

3 $$\frac{{S}_{ik}^{j^{\prime} }-2}{3-2}=\frac{{S}_{ik}^{j}-{X}_{3}}{{X}_{4}-{X}_{3}}$$

#### Aggregation method

With the area-weighted normalization and scaling transformation, the scaling normalization score of 14 security evaluation indicators at every grid ${S}_{ik}^{j^{\prime} }$ was calculated. Two aggregation strategies, including the veto aggregation and the balanced aggregation methods^28, 50^, were used to comprehensively integrate evaluation scores of multiple indicators. The veto aggregation method focuses on the most serious security deficits of all indicators, which could help find the limit factor of ecological and environmental security. The equation is as follows:

4 $${V}_{i}^{j}=\underset{i=1}{\overset{n}{{\rm{\max }}}}\{{S}_{ik}^{j^{\prime} }\}$$

where ${V}_{i}^{j}$ is the score calculated by the veto aggregation method; n is the number of security evaluation indicators.

The balanced aggregation method coordinates all indicators and calculates the mean value of indicator scores by the following equation:

5 $${G}_{i}^{j}=\sum _{i=1}^{n}{w}_{i}\times {S}_{ik}^{j^{\prime} }$$

where ${G}_{i}^{j}$ is the score calculated by the balanced aggregation method; *w*_*i*_ denotes the weight of security evaluation indicator, which is equal to 1/*n*.

Using the veto aggregation and the balanced aggregation methods, we evaluated the scores of water security, land security, air security and biodiversity security. Then, the water, land, air, and biodiversity security scores were grouped into six classes (class_1_: safety; class_2_: basic safety; class_3_: endangered security; class_4_: insecurity; class_5_: marginal crisis; class_6_: crisis) in ArcGIS 10.1 (ESRI Inc., USA) by Jenks natural break optimization for the balanced aggregation scores and the uniform interval ranges of [1.0, 1.5], (1.5, 2], (2, 3], (3, 4], (4, 5], (5, 6] for the veto aggregation scores. The uniform classes of the veto aggregation method could be easily interpreted based on the indicator classified standard. For example, if the score of the security evaluation is larger than 5.00, it implies that there is at least one security evaluation indicator up to the crisis level, which indicates a serious security status.

The final goal of ecological and environmental security was evaluated by aggregating four aspects of securities including 14 indicators. The final security scores were calculated by two aggregation methods by using the following equations:

6 $$E{V}_{i}^{j}=\,\max \,\{W{V}_{i}^{j},L{V}_{i}^{j},A{V}_{i}^{j},B{V}_{i}^{j}\}$$

7 $$E{G}_{i}^{j}=\frac{1}{4}\times (W{G}_{i}^{j}+L{G}_{i}^{j}+A{G}_{i}^{j}+B{G}_{i}^{j})$$

where $E{V}_{i}^{j}$ and $E{G}_{i}^{j}$ are the ecological and environmental security evaluation scores by the veto aggregation and balanced aggregation methods, respectively; $W{V}_{i}^{j}$, $L{V}_{i}^{j}$, $A{V}_{i}^{j}$, and $B{V}_{i}^{j}$ are the water, land, air, and biodiversity security scores calculated using the veto aggregation method, respectively; $W{G}_{i}^{j}$, $L{G}_{i}^{j}$, $A{G}_{i}^{j}$, $B{G}_{i}^{j}$ are the water, land, air, and biodiversity security scores calculated using the balanced aggregation method, respectively. Moreover, the non-evaluation areas were classified to the class of “crisis” in the final evaluation of the ecological and environmental security. The ecological and environmental security scores were grouped into six classes in ArcGIS 10.1 by Jenks natural break optimization. Spatial distributions were visualized in ArcGIS 10.1.

## Footnotes

1 Report from Ministry of Environmental Protection of the People’s Republic of China, 2017 (in Chinese).

2 Ministry of Water Resources of the People’s Republic of China, 1997, SL190-96 Standards for Grading of Soil Erosion. (in Chinese).
